# Injuries according to sexual maturity status: a three-season observational study with male academy players of a professional Spanish football club

**DOI:** 10.5114/biolsport.2026.154143

**Published:** 2025-09-29

**Authors:** Mauricio Monaco, Eirik Halvorsen Wik, Abdulaziz Farooq, Gil Rodas

**Affiliations:** 1 FC Barcelona Medical Department, FIFA Medical Excellence Center, Barcelona, Spain; 2 Aspetar Orthopaedic & Sports Medicine Hospital, Doha, Qatar; 3 Institute of Sport and Exercise Medicine, Department of Exercise, Sport and Lifestyle Medicine, Faculty of Medicine and Health Sciences, Stellenbosch University, Stellenbosch, South Africa; 4 Barça Innovation Hub, Health & Wellness Area, Barcelona, Spain

**Keywords:** Adolescent, Epidemiology, Puberty, Athletic Injuries, Growth and Development, Tanner Stage, Sexual Maturity, Football Injuries

## Abstract

The current literature suggests that football (soccer) players’ age and maturity status affect the likelihood of sustaining certain injuries; however, few studies have used indicators of sexual maturity. By retrospectively analysing prospectively collected data, we therefore aimed to describe injury patterns among young football players in a Spanish academy over three seasons and investigate associations with sexual maturity status. Participants included 354 male youth players aged 7 to 18, categorised into five age groups. Maturity assessments were conducted annually, utilising pubertal stages for genitalia and pubic hair, alongside testicular volume measurements. Time-loss injuries were recorded by medical staff and associations with pubertal stages were examined using Generalized Estimating Equations (GEE). Results indicated an overall incidence of 2.2 injuries per 1000 hours. Higher injury rates were observed for more advanced maturational stages (p < 0.05). Common injury types included muscle injuries (23%), joint sprains (20%), and growth-related injuries (16%), with specific injuries varying by maturity stage. For instance, growth-related injuries peaked during mid-puberty, while muscle and joint injuries were more frequent in advanced maturity stages (p < 0.05). Our findings suggest that sexual maturity status was significantly associated with injury occurrence in youth football. This emphasises the importance of understanding the interplay between biological maturity and injury occurrence. In addition to considering players’ age or playing level, coaches and clinicians may consider tailoring training and injury prevention strategies according to players’ maturity levels to better mitigate injury risks.

## INTRODUCTION

Puberty represents the transition between childhood and adulthood [1], including major physical, psychological and behavioural changes. Although the current body of research is not conclusive, athletes’ maturity status (i.e., how far an athlete has progressed through this transition to adulthood or the mature state) [1] appears to be relevant in terms of how many and which (e.g., location and type) injuries they sustain [2, 3, 4]. Better understanding how injury patterns differ according to maturational stages may, therefore, aid clinicians and practitioners in reducing injury occurrence in young athletes.

Football injuries have been widely studied in the context of growth and maturation, but most prospective investigations have used indirect approaches (e.g., somatic maturity by equations based on anthropometric measures) to estimate maturation during puberty [4], leaving the link between indicators of sexual maturity and injury risk unclear. Assessment of sexual maturation is an established method for determining the maturity status of individuals, commonly through direct observation of secondary sex characteristics such as pubic hair, genital or breast development in a clinical setting [1, 5, 6]. Individuals can subsequently be classified into specific pubertal stages, often using the criteria developed by Tanner in 1962.^7^ In boys, assessments of testicular volume are useful to rule out pathologies and identify the onset of puberty [1, 5]. The use of secondary sex characteristics in periodic medical examinations can be considered valuable due to the close relationship between these indicators and circulating hormones, low cost of assessments, reproducibility, and ability to simultaneously identify medical conditions and assess biological maturation [1, 8, 9, 10, 11, 12]. Indicators of sexual maturity have been used to contextualise the occurrence of pain, injuries (e.g., anterior cruciate ligament tears) and their potential risk factors in large-scale studies in the general population [13, 14, 15]. Few studies have reported the relationship between sexual maturity status and prospective injury in sports [12, 16, 17, 18, 19, 20, 21, 22], perhaps because these assessments can be considered invasive, especially in non-clinical settings [23]. Although some of these suggest a potential relationship between athletes’ pubertal stages and the occurrence of certain injury types, the variation in study design and the low number of studies in football specifically limit our ability to draw firm conclusions.

Despite the potential for information on sexual maturity status to provide important information about players’ predisposition to injury, data in support of this is lacking in high-level youth football players. The primary aim of this study was therefore to describe injury occurrence according to sexual maturity status using data collected routinely over three seasons in the academy of a Spanish professional football club.

## MATERIALS AND METHODS

This study is a retrospective review of prospectively collected data. Data were collected during the 2010/11 through 2012/13 seasons in one professional club’s football academy in Catalonia, Spain, as part of their routine annual medical screening and injury surveillance programme.

### Participants

Eligible participants were healthy male football players aged 7 through 18 years, who trained and competed in five separate age groups (U10, U12, U14, U16 and U19). Only data from players who attended the annual medical screening were considered for analyses, excluding players who were not part of their team at the start of the competitive season (e.g., players who joined the club later). Teams typically trained once per day from Monday through Thursday and played one game at the weekend. As part of the annual medical screening, written informed consent to use data from routine club screening and monitoring practices for research purposes was obtained from parents, while players assented to data collection procedures; this decision did not affect their opportunity to participate in the academy program. Ethical approval to retrospectively analyse data stored in the club database for this study was obtained from the local research ethics committee (Science and Ethics of the Barca Innovation Hub, FC Barcelona, ref no.: n*2017FCB12), and the study was approved by the FC Barcelona Medical Committee. The study conformed to the recommendations of the Declaration of Helsinki.

### Assessments of maturity

Maturity assessments were routinely carried out during the annual paediatric medical screening at the start of each season (i.e., they were not included specifically for research purposes). Screenings were in accordance with local government guidelines [24] and international recommendations [6, 25] including pubertal staging by genitalia and pubic hair (prepubertal state: G1/PH1, early puberty: G2/PH2, mid-puberty: G3/PH3 and G4/PH4 and as mature: G5/PH5-PH6) using criteria from a comparative atlas [7, 26] and a measurement of testicular volume using a Prader orchidometer (1 to 25 ml) [27]. Categories for testicular volume were created after collecting the data, separating four distinct phases: “prepubertal” (TV1: 1, 2 and 3 ml), “initial or onset of puberty” (TV2: 4, 5 and 6 ml), “mid-puberty” (TV3: 8, 10 and 12 ml) and “advanced or end of puberty” (TV4: 15, 20 and 25 ml) [12]. Maturity assessments were completed by the same (i.e., one) experienced paediatrician and players or the parents could choose not to participate. This decision did not affect the player’s status within the academy programme.

### Injury surveillance

Injuries and illnesses were recorded continuously to an electronic medical record throughout the observation period by three experienced sports medicine physicians, following the consensus guidelines described by Fuller et al. [28]. A time-loss definition was used, i.e., only injuries that led to a player missing a minimum of one football session were included. A diagnosis code was provided for all entries based on the Orchard Sports Injury Classification System (OSICS) Version 10 [29] alongside information about injury circumstances and duration. Clinicians had access to imaging facilities (e.g., radiography, computed tomography (CT), magnetic resonance imaging (MRI), ultrasound) where indicated, improving the accuracy of injury diagnoses. Following the completion of data collection, “Medical” diagnoses that were not injuries (Tier One code “M”) were removed, while “Paediatric” injuries (Tier One code “J”) were re-classified and assigned to the most appropriate anatomical site by one medical doctor/researcher. This group was named “growth-related injuries” (n = 63) and included 59 diagnoses of apophysitis/avulsion (anterior inferior iliac spine (AIIS) n = 20; anterior superior iliac spine (ASIS) n = 10; Osgood-Schlatter disease n = 7; ischial tuberosity n = 7; groin n = 5; iliac crest n = 5; Sinding-Larsen-Johansson disease n = 3; Sever’s disease n = 1; 5^th^ metatarsal n = 1), two diagnoses of epiphysitis (medial tibial plateau n = 1; distal radius n = 1) and two diagnoses of osteochondrosis (wrist/hand n = 1; foot n = 1). Individual football exposure was recorded separately by club technical staff, based on continuous documentation of player attendance and session duration.

### Statistical analyses

Data were coded and analysed using SPSS software v21.0. Summary statistics are presented as mean and standard deviation (SD) or median and interquartile range (IQR) for continuous variables, whereas frequencies and proportions were used to summarise categorical variables. Injury incidence was calculated as the number of injuries per 1000 hours of exposure. Generalised estimating equations (GEE) were used to account for repeated observations of the same athletes in multiple seasons. Injury counts were entered as the dependent variable of interest, with log-transformed exposure used as the offset variable. The link function selected was a Poisson distribution, using logit and injury incidence with a 95% confidence interval (CI). The above analysis was repeated to add categorical factors of interest – age group, pubic hair stage, genital stage and testicular volume category – separately to examine differences in injury incidence. Injury burden was computed as days of absence per 1000 player-hours. Poisson 95% CI were calculated [30], and differences between burden and injury rates were calculated using techniques described by Frome and Checkoway [31]. Since age was considered a potential confounding variable for injury occurrence, we also computed age-adjusted injury incidence for all maturity variables. Furthermore, the same GEE analysis procedure was used to compute injury incidence for injury locations and types, where maturity variables were included in the model to determine their association with injury. A p-value < 0.05 was used as a threshold for statistical significance.

## RESULTS

### Participants and exposure

In total, 354 football players (mean age 12.3 years, SD 3.0, range 7 to 18) were included in the study, completing 670 player-seasons (one season: 145 players, two seasons: 102 players, three seasons: 107 players). Players accumulated 174,597 hours of football exposure during the observation period, equating to 261 hours per player per year on average. The total number of players, average age, mean player hours, and distribution of players across age groups were similar across the three seasons (Table 1).

**TABLE 1:** Injury incidence, severity and burden by age group and sexual maturity status in Spanish male academy football players.

				Injury/1000 h (95% CI)		Severity of time loss injuries Median [IQR]	Burden (days lost/1000 h) (95% CI)

	2010–11	2011–12	2012–13	Overall	Age-adjusted

Players	211	219	240				

Mean age (SD)	12.4 (2.8)	12.2 (3.0)	12.3 (3.0)				

Mean player hours (SD)	257.1 (46.6)	260.2 (50.7)	264.1 (47.5)				

Time-loss injuries	133	113	142	2.2 (2.0–2.5)	1.99 (1.8–2.2)	16.0 [7.0–37.0]	55.0 (53.9–56.1)
U10	37	46	47	1.1 (0.8–1.6)	0.8 (0.4–1.6)	10.5 [7.0–26.0]	16.5 (15.0–18.1)
U12	48	50	53	1.6 (1.2–2.1)	1.4 (1.0–2.0)	14.0 [7.0–28.0]	24.5 (22.9–26.3)^B^
U14	43	42	50	1.6 (1.3–2.1)	1.6 (1.3–2.1)	14.0 [7.0–25.0]	35.7 (33.8–37.7)^B,A^
U16	44	39	42	3.4 (2.8–4.0) ^B,A,I^	3.9 (2.8–5.4) ^B,A,I^	23.0 [8.0–53.0] ^B,A,I^	98.0 (94.8–101.3)^B,A,I,J^
U19	39	42	48	3.0 (2.5–3.6) ^B,A,I^	4.0 (2.2–7.3)	20.0 [8.0–55.0] ^B,A,I^	83.8 (81.1–86.7)^B,A,I^

Players with maturity assessment^#^	194	197	204				
G1 (%)	(38.1)	(45.7)	(47.5)	1.5 (1.2–1.8)	1.9 (1.4–2.5)	10.0 [7.0–20.0]	20.6 (19.5–21.9)
G2 (%)	(10.8)	(10.2)	(8.8)	1.4 (0.9–2.1)	1.4 (0.9–2.2)	14.0 [7.0–24.0]	22.4 (20.0–24.9)
G3 (%)	(14.9)	(7.6)	(9.3)	1.9 (1.4–2.7)	1.7 (1.2–2.5)	25.0 [14.0–38.0]^1^	49.3 (46.0–52.8)^1,2^
G4 (%)	(12.4)	(8.1)	(11.3)	2.8 (2.1–3.7)^1,2^	2.3 (1.7–3.2)	20.0 [12.0–56.0]^1^	90.8 (86.3–95.3)^1,2,3,5^
G5 (%)	(23.7)	(28.4)	(23.0)	3.1 (2.7–3.7)^1,2,3^	2.1 (1.5–3.0)	21.0 [7.0–53.0]^1^	85.1 (82.4–87.9)^1,2,3^

PH1 (%)	(41.8)	(50.3)	(51.0)	1.5 (1.2–1.8)	1.8 (1.4–2.3)	10.0 [7.0–21.5]	21.5 (20.4–22.7)
PH2 (%)	(7.7)	(6.6)	(5.4)	1.3 (0.7–2.2)	1.3 (0.7–2.3)	14.0 [10.0–24.0]	19.0 (16.3–22.0)
PH3 (%)	(13.4)	(5.1)	(9.3)	1.9 (1.3–2.8)	1.7 (1.2–2.5)	21.0 [5.0–38.0]	50.9 (47.3–54.6)^1.2^
PH4 (%)	(11.9)	(10.2)	(11.3)	2.6 (1.9–3.4)	2.2 (1.6–3.0)	20.0 [12.0–44.0]^1^	56.7 (53.3–60.3)^1,2,3^
PH5 (%)	(20.1)	(24.4)	(15.7)	3.2 (2.7–3.9)^1,2^	2.3 (1.6–3.0)	23.5 [7.0–54.0]^1^	91.0 (87.9–94.3)^1,2,3,4^
PH6 (%)	(5.2)	(3.6)	(7.4)	3.3 (2.3–4.7)^1,2^	2.3 (1.4–3.7)	17.5 [6.0–40.0]	123.3 (116.2–130.7)^1,2,3,4,5^

TV1 (%)	(34.5)	(46.2)	(48.0)	1.4 (1.2–1.8)	1.8 (1.3–2.3)	10.0 [6.0–20.0]	20.9 (19.7–22.1)
TV2 (%)	(12.4)	(10.7)	(10.3)	1.6 (1.0–2.3)	1.6 (1.1–2.4)	14.0 [7.0–24.5]	23.7 (21.4–26.2)^1^
TV3 (%)	(14.4)	(12.2)	(13.7)	1.6 (1.2–2.2)	1.4 (1.0–2.0)	21.0 [14.0–31.0]^1^	50.9 (47.9–54.0)^1,2^
TV4 (%)	(38.7)	(31.0)	(27.9)	3.2 (2.8–3.7)^1,2,3^	2.4 (1.8–3.3)	21.0 [7.0–53.5]^1^	89.3 (86.8–91.8)^1,2,3^

* Includes only injuries for players with maturity assessment.

G1-5: Genital stage 1–5; PH1-6: Pubic hair stage 1–6; TV1-4: Testicular volume stage 1–4.

Characters in superscript indicate significant differences (p < 0.05) compared to other stages within the same group. Superscript letters indicate statistically significant differences (p < 0.05) compared to the corresponding age group (^B^U10, ^A^U12, ^I^U14, ^C^U16, ^j^U19) or pubertal stage (^1^Stage 1, ^2^Stage 2, ^3^Stage 3, ^4^Stage 4, ^5^Stage 5).

### Maturity assessments

**FIG. 1:**
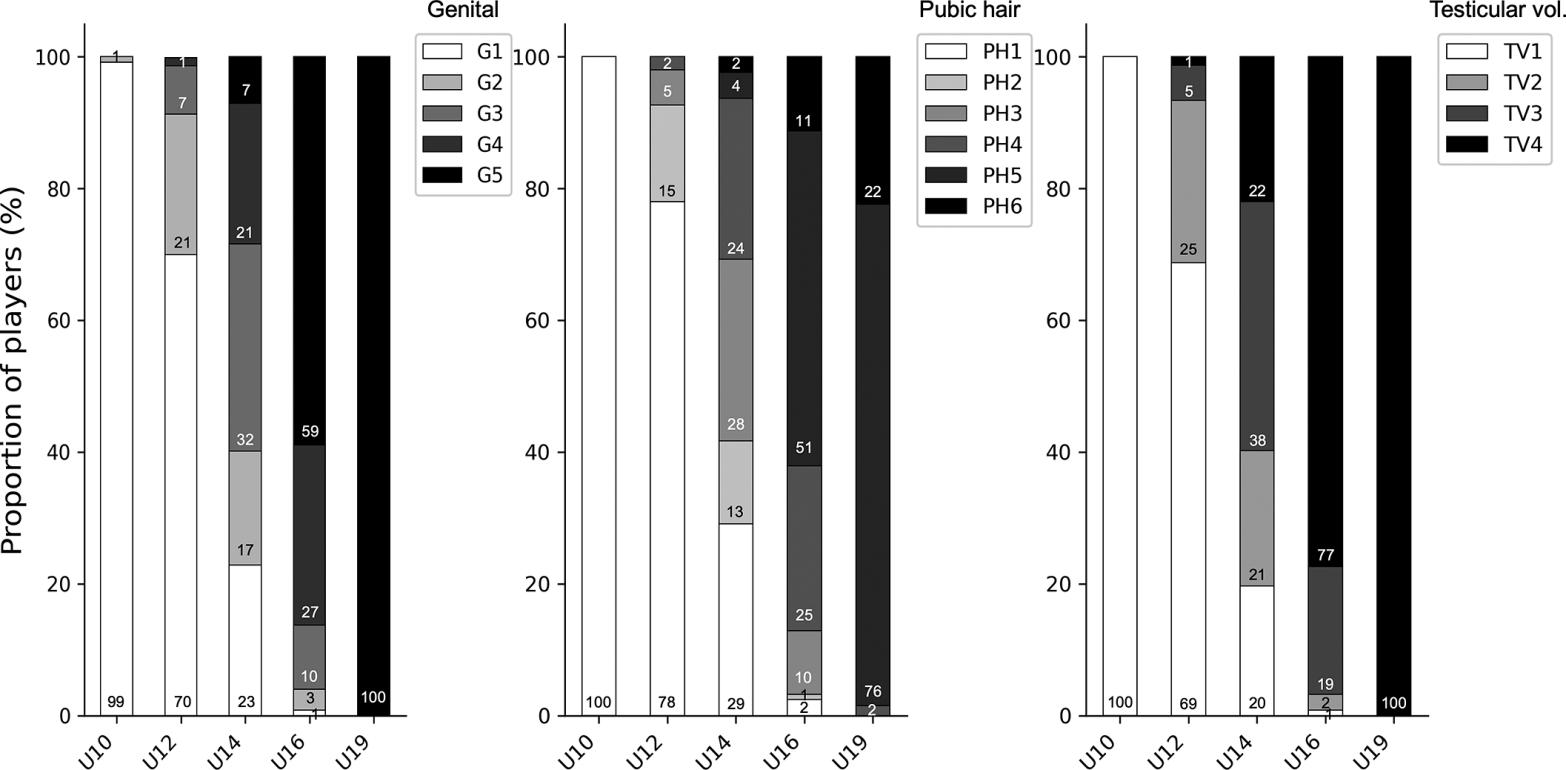
Distribution (%) of players at different stages of maturity within age groups in a Spanish male football academy.

Maturity assessments were available for 595 player-seasons (89% of the total; excluded 17/211 players for 2011/12, 22/219 players for 2012/13 and 36/240 players for 2012/13). The proportion of players classified to each maturity stage is presented per season in Table 1 and within each age group in Figure 1. Across the different maturity indicators, almost all U10 players were in G1, PH1 and TV1 and almost all U19 players in G5, PH5/6 and TV4, with more diversity observed in the U12, U14 and U16 age groups.

### Injury occurrence

Considering all academy players, 388 time-loss injuries were recorded during the observation period, with 44% of players sustaining at least one injury. Seven injuries could be classified as subsequent local injuries as they were to the same location and side as a previous injury, but had a different diagnosis (three of these occurred within one month of returning from the previous event). The overall injury incidence was 2.2 per 1000 hours (95% CI: 2.0 to 2.5), with similar rates for all three seasons. The three most common injury locations were the thigh (21%), pelvis & groin (20%) and ankle (17%), while the most common injury types were muscle injuries (23%), joint sprains (20%) and growth-related injuries (16%). Injury incidence was significantly greater in the two most advanced age groups compared to the three younger age groups (Table 1). Significant differences between age groups were also observed when grouped by body part (Table 2) and injury types (Table 3).

**TABLE 2:** Injury incidence (injuries per 1000 hours; 95% CI), for the most relevant injury locations in a Spanish male football academy, by age group and sexual maturity status.

	Foot	Ankle	Lower leg	Knee	Thigh	Pelvis & Groin	Wrist & Hand	Head
Injury count	23	67	20	60	80	77	23	14

Overall incidence	0.1 (0.1–0.2)	0.4 (0.3–0.5)	0.1 (0.1–0.2)	0.3 (0.3–0.4)	0.5 (0.4–0.6)	0.3 (0.4–0.6)	0.1 (0.1–0.2)	0.1 (0.0–0.1)
U10	0.2 (0.1–0.4)	0.1 (0.0–0.3)	0.0 (0.0–0.0)	0.1 (0.0–0.3)	0.2 (0.1–0.5)	0.2 (0.1–0.4)	0.1 (0.0–0.3)	0.1 (0.0–0.3)
U12	0.1 (0.1–0.3)	0.2 (0.1–0.4)	0.1 (0.0–0.3)	0.3 (0.1–0.5)	0.3 (0.1–0.5)	0.3 (0.2–0.6)	0.3 (0.1–0.5) ^I,J^	0.0 (0.0–0.2)
U14	0.1 (0.0–0.3)	0.2 (0.1–0.4)	0.1 (0.0–0.3) ^B^	0.2 (0.1–0.4)	0.2 (0.1–0.4)	0.5 (0.3–0.8) ^B,J^	0.1 (0.0–0.2)	0.0 (0.0–0.2)
U16	0.2 (0.1–0.5)	0.5 (0.3–0.8) ^A,B^	0.1 (0.0–0.3) ^B^	0.4 (0.3–0.7) ^B^	0.7 (0.5–1.0) ^A,B,I^	1.0 (0.7–1.4) ^A,B,I,J^	0.2 (0.1–0.4)	0.1 (0.0–0.3)
U19	0.1 (0.0–0.2)	0.8 (0.6–1.1) ^A,B,I^	0.2 (0.1–0.4) ^B^	0.6 (0.4–0.9) ^A,B,I^	0.8 (0.6–1.2) ^A,B,I^	0.2 (0.1–0.4)	0.1 (0.0–0.2)	0.1 (0.1–0.3)

Injury count^*^	21	55	13	45	56	75	22	11
G1	0.2 (0.1–0.3) ^2^	0.2 (0.8–0.3)	0.1 (0.1–0.2)	0.2 (0.1–0.4)	0.2 (0.1–0.4)	0.3 (0.2–0.5)	0.1 (0.1–0.2)	0.1 (0.0–0.2)
G2	0.0 (0.0–0.0)	0.1 (0.0–0.5)	0.0 (0.0–0.0)	0.3 (0.1–0.7)	0.3 (0.1–0.7)	0.3 (0.1–0.7)	0.3 (0.1–0.7)	0.1 (0.0–0.5)
G3	0.1 (0.0–0.4)	0.1 (0.0–0.5)	0.1 (0.0–0.4)	0.2 (0.1–0.6)	0.2 (0.1–0.6)	0.6 (0.3–1.1)	0.2 (0.1–0.6)	0.1 (0.0–0.5)
G4	0.2 (0.1–0.5)	0.6 (0.4–1.1) ^1,2,3^	0.0 (0.0–0.0)	0.4 (0.2–0.8)	0.4 (0.2–0.8)	1.0 (0.7–1.7) ^1,2^	0.1 (0.0–0.5)	0.0 (0.0–0.0)
G5	0.2 (0.1–0.3) ^2^	0.7 (0.5–1.1) ^1,2,3^	0.1 (0.1–0.3) ^2,4^	0.5 (0.3–0.7) ^1^	0.7 (0.5–1.0) ^1,2,3^	0.6 (0.4–0.9)	0.1 (0.1–0.3)	0.1 (0.1–0.3) ^4^

PH1	0.2 (0.1–0.3) ^2^	0.1 (0.1–0.3)	0.1 (0.0–0.2) ^2,4^	0.2 (0.1–0.4)	0.2 (0.1–0.4)	0.3 (0.2–0.5)	0.1 (0.1–0.3)	0.1 (0.0–0.2) ^2^
PH2	0.0 (0.0–0.0)	0.1 (0.0–0.8)	0.0 (0.0–0.0)	0.2 (0.1–0.8)	0.2 (0.1–0.8)	0.4 (0.2–1.1)	0.2 (0.1–0.8)	0.0 (0.0–0.0)
PH3	0.1 (0.0–0.5)	0.1 (0.0–0.5)	0.1 (0.0–0.5)	0.2 (0.1–0.6)	0.2 (0.1–0.6)	0.6 (0.3–1.2)	0.3 (0.1–0.7)	0.1 (0.0–0.5)
PH4	0.2 (0.1–0.5)	0.7 (0.4–1.2) ^1,2,3^	0.0 (0.0–0.0)	0.3 (0.2–0.7)	0.3 (0.2–0.7)	0.8 (0.5–1.4) ^1^	0.1 (0.0–0.4)	0.1 (0.0–0.4)
PH5	0.2 (0.1–0.4) ^2^	0.6 (0.4–1.0) ^1,2,3^	0.2 (0.1–0.4) ^2,4^	0.5 (0.3–0.8) ^1^	0.7 (0.5–1.1) ^1,2,3^	0.6 (0.4–1.0) ^1^	0.2 (0.1–0.4)	0.1 (0.00–0.3) ^2^
PH6	0.2 (0.1–0.9)	1.0 (0.5–1.9) ^1,2,3^	0.1 (0.0–0.8)	0.3 (0.1–1.0)	0.6 (0.2–1.3)	0.8 (0.4–1.6)	0.1 (0.0–0.8)	0.1 (0.0–0.8)

TV1	0.2 (0.1–0.3) ^2^	0.2 (0.1–0.3)	0.1 (0.0–0.2)	0.2 (0.1–0.4)	0.2 (0.1–0.4)	0.3 (0.2–0.5)	0.1 (0.1–0.2)	0.1 (0.0–0.2)
TV2	0.0 (0.0–0.0)	0.1 (0.0–0.5)	0.1 (0.0–0.5)	0.3 (0.1–0.8)	0.3 (0.1–0.7)	0.4 (1.2–0.8)	0.2 (0.1–0.6)	0.1 (0.0–0.4)
TV3	0.1 (0.0–0.3)	0.1 (0.0–0.4)	0.1 (0.0–0.3)	0.2 (0.1–0.5)	0.1 (0.0–0.4)	0.6 (0.4–1.0)	0.2 (0.1–0.5)	0.0 (0.0–0.0)
TV4	0.2 (0.1–0.3) ^2^	0.8 (0.6–1.0) ^1,2,3^	0.1 (0.1–0.2)	0.4 (0.3–0.7) ^1,3^	0.7 (0.5–0.9) ^1,2,3^	0.7 (0.5–1.0) ^1^	0.2 (0.1–0.3)	0.1 (0.1–0.3) ^3^

* Includes only injuries for players with maturity assessment.

G1-5: Genital stage 1–5; PH1-6: Pubic hair stage 1–6; TV1-4: Testicular volume stage 1–4.

Characters in superscript indicate significant differences (p < 0.05) compared to other stages within the same group. Superscript letters indicate statistically significant differences (p < 0.05) compared to the corresponding age group (^B^U10, ^A^U12, ^I^U14, ^C^U16, ^j^U19) or pubertal stage (^1^Stage 1, ^2^Stage 2, ^3^Stage 3, ^4^Stage 4, ^5^Stage 5).

**TABLE 3:** Injury incidence (injuries per 1000 hours; 95% CI), for the most relevant injury types in a Spanish male football academy, by age group and sexual maturity status.

	Muscle injury	Joint sprain	Growth-related	Fracture	Hematoma/Bruising	Tendon injury
Injury count	90	76	63	34	28	23

Overall incidence	0.5 (0.4–0.6)	0.4 (0.4–0.6)	0.4 (0.3–0.5)	0.2 (0.1–0.3)	0.2 (0.1–0.2)	0.1 (0.1–0.2)

U10	0.2 (0.1–0.5)	0.2 (0.1–0.4)	0.2 (0.1–0.4)	0.2 (0.1–0.5)	0.0 (0.0–0.3)	0.0 (0.0–0.3)
U12	0.3 (0.1–0.5)	0.2 (0.1–0.4)	0.4 (0.3–0.7) ^J^	0.2 (0.1–0.5)	0.1 (0.0–0.3)	0.1 (0.0–0.3)
U14	0.2 (0.1–0.5)	0.2 (0.1–0.4)	0.6 (0.4–0.9) ^B,J^	0.2 (0.1–0.4)	0.2 (0.1–0.4)	0.1 (0.0–0.2)
U16	0.8 (0.5–1.1) ^A,B,I^	0.7 (0.4–1.0) ^A,B,I^	0.6 (0.4–0.9) ^B,J^	0.3 (0.1–0.5)	0.3 (0.2–0.6) ^A,B^	0.3 (0.1–0.5) ^B,I^
U19	0.9 (0.7–1.3) ^A,B,I^	0.9 (0.6–1.2) ^A,B,I^	0.1 (0.0–0.2)	0.1 (0.1–0.3)	0.2 (0.1–0.4)	0.2 (0.1–0.4) ^B^

Injury count^*^	63	61	62	30	24	19
G1	0.2 (0.1–0.4)	0.2 (0.1–0.4)	0.4 (0.3–0.6)	0.1 (0.1–0.3)	0.1 (0.1–0.2)	0.1 (0.0–0.2)
G2	0.4 (0.1–0.8)	0.1 (0.0–0.6)	0.3 (0.1–0.7)	0.2 (0.1–0.6)	0.2 (0.1–0.6)	0.1 (0.0–0.5)
G3	0.3 (0.1–0.7)	0.1 (0.0–0.4)	0.8 (0.5–1.3) ^5^	0.4 (0.2–0.8)	0.2 (0.1–0.6)	0.1 (0.0–0.4)
G4	0.4 (0.2–0.9)	0.5 (0.3–1.0) ^3^	0.6 (0.4–1.1)	0.2 (0.1–0.6)	0.3 (0.1–0.7)	0.1 (0.0–0.5)
G5	0.8 (0.6–1.1) ^1,2,3^	0.9 (0.6–1.2) ^1,2,3^	0.3 (0.1–0.5)	0.2 (0.1–0.4)	0.2 (0.1–0.3)	0.3 (0.2–0.5) ^1,2,3^

PH1	0.3 (0.2–0.4)	0.2 (0.1–0.4) ^2^	0.4 (0.2–0.6)	0.2 (0.1–0.3)	0.1 (0.1–0.2)	0.1 (0.0–0.2)
PH2	0.2 (0.1–0.8)	0.0 (0.0–0.0)	0.4 (0.2–1.1)	0.2 (0.1–0.8)	0.2 (0.1–0.8)	0.1 (0.0–0.8)
PH3	0.3 (0.1–0.7)	0.1 (0.0–0.5)	0.8 (0.5–1.4) ^5,6^	0.3 (0.1–0.8)	0.1 (0.0–0.5)	0.1 (0.0–0.5)
PH4	0.3 (0.1–0.7)	0.5 (0.3–1.0) ^2,3^	0.6 (0.3–1.1)	0.3 (0.1–0.7)	0.4 (0.2–0.8)	0.1 (0.0–0.4)
PH5	0.9 (0.6–1.3) ^1,2,3,4^	0.8 (0.5–1.1) ^1,2,3^	0.3 (0.2–0.5)	0.2 (0.1–0.4)	0.2 (0.1–0.4)	0.3 (0.1–0.5) ^1^
PH6	0.7 (0.3–1.5)	1.3 (0.8–2.3) ^1,2,3,4^	0.2 (0.1–0.9)	0.1 (0.0–0.8)	0.1 (0.0–0.8)	0.2 (0.1–0.9)

TV1	0.2 (0.1–0.4)	0.2 (0.1–0.4)	0.4 (0.3–0.6)	0.1 (0.1–0.3)	0.1 (0.0–0.2)	0.1 (0.0–0.2)
TV2	0.3 (0.1–0.8)	0.2 (0.1–0.6)	0.4 (0.2–0.8)	0.1 (0.3–0.5)	0.3 (0.1–0.7)	0.1 (0.0–0.5)
TV3	0.2 (0.1–0.5)	0.1 (0.0–0.4)	0.6 (0.4–1.1)	0.4 (0.2–0.8)	0.1 (0.0–0.3)	0.1 (0.0–0.4)
TV4	0.8 (0.6–1.0) ^1,2,3^	0.8 (0.6–1.1) ^1,2,3^	0.4 (0.2–0.5)	0.2 (0.1–0.4)	0.3 (0.2–0.4) ^1,3^	0.2 (0.1–0.4) ^1^

* Includes only injuries for players with maturity assessment.

G1-5: Genital stage 1–5; PH1-6: Pubic hair stage 1–6; TV1-4: Testicular volume stage 1–4.

Characters in superscript indicate significant differences (p < 0.05) compared to other stages within the same group. Superscript letters indicate statistically significant differences (p < 0.05) compared to the corresponding age group (^B^U10, ^A^U12, ^I^U14, ^C^U16, ^j^U19) or pubertal stage (^1^Stage 1, ^2^Stage 2, ^3^Stage 3, ^**4**^Stage 4, ^5^Stage 5).

**FIG. 2:**
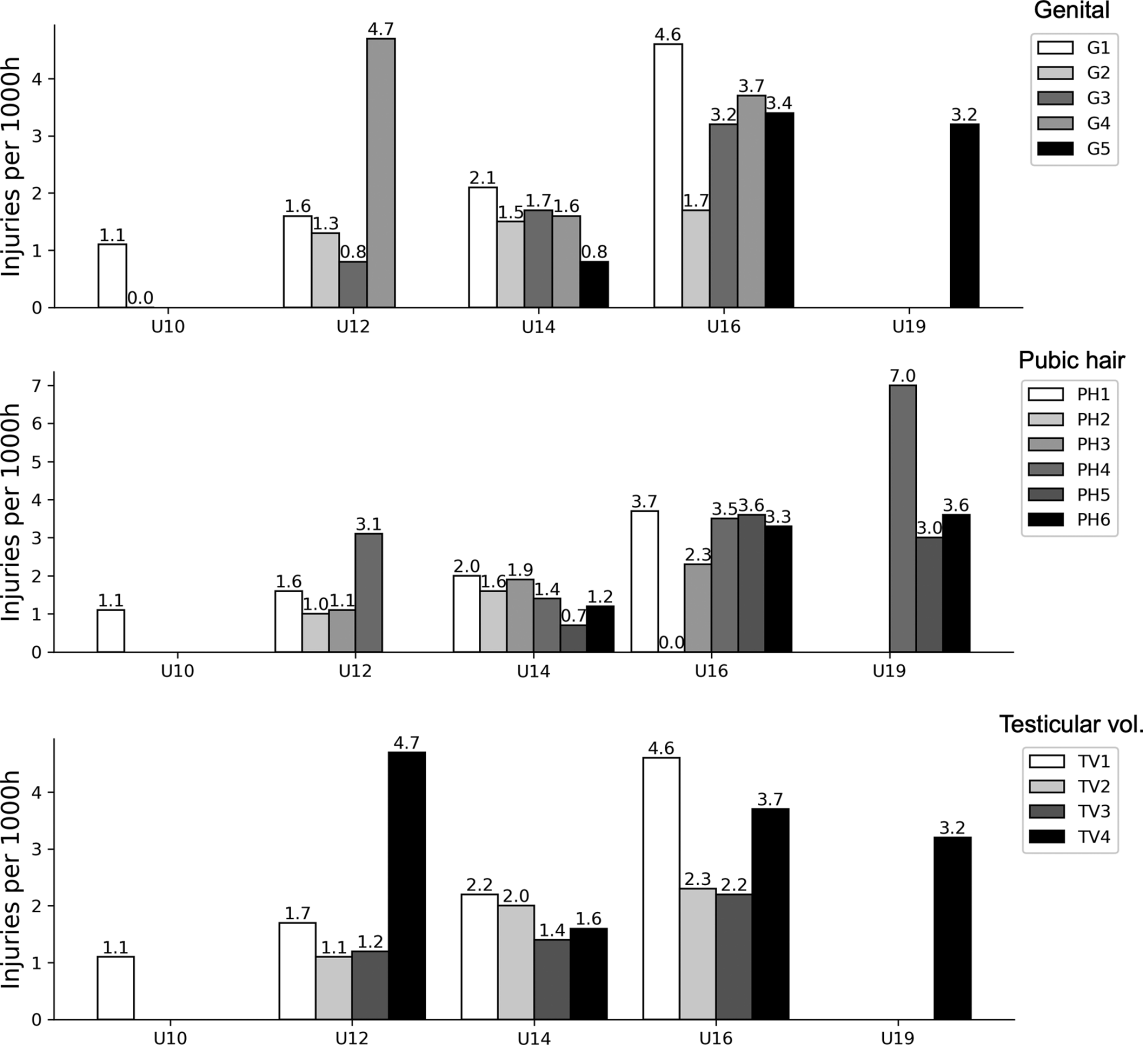
Injury incidence (injuries per 1000h, 95% CI) for different stages of sexual maturity within age groups of a Spanish male football academy.

When only considering players with a maturity assessment, 319 time-loss injuries were included for analyses. Combining all age groups, a significantly greater overall incidence was observed at more advanced stages of maturity (G4-5, PH5-6 and TV4), with no significant between-group differences after adjusting for player age (Table 1). Injury incidence for different maturity stages within each age group is presented in Figure 2. When examining injury location, ankle, knee, thigh, and pelvis & groin injuries were significantly more common at more advanced stages of maturation (Table 2). More mature players sustained more muscle injuries, joint sprains, hematomas/bruising and tendon injuries compared to those who were less mature, while players in mid-puberty (G3 and PH3) had a significantly greater incidence of growth-related injuries compared to more mature players (Table 3). Additional age-adjusted analyses for location and type per maturity stage are included in Supplementary Table 1 and Table 2; in this study, we observed a significantly greater incidence of joint sprains for players in PH6 compared to PH2.

## DISCUSSION

Our primary aim was to describe injury incidence – overall, by location and by type – according to age group and sexual maturity status in a cohort of male academy football players. Using assessments that formed part of routine clinical practice at one professional Spanish club, we observed an expected gradual shift in maturity stage distribution from younger to older age groups and a higher overall injury incidence with increased maturity. Certain injury locations (ankle, thigh, knee and pelvis/groin) were more common in more mature players. Muscle injuries, joint sprains, tendon injuries and hematomas/bruising were also more frequent at advanced stages of puberty, while growth-related injuries were more common in players who were classified as mid-pubertal.

### A wide range of pubertal stages was observed in U12, U14 and U16 age groups

As expected, the distribution of players across different stages of sexual maturity gradually changed from the youngest to the oldest age groups. The U14 age group was the most diverse, whilst the U10 (mainly prepubertal) and U19 (mainly advanced/end-maturity) age groups consisted of more homogeneous groups of players. Our findings are in line with observations of British adolescent males by Marshall and Tanner [32] who reported that the onset of puberty could be expected at any age between 9.5 and 13.5 years, with an expected age range from 13 to 17 years for reaching maturity. Furthermore, they stated that any stage of genital development could be expected between the ages of 13 and 15 years [32], which was also the case in our sample. The variation in maturity status within age groups in our study is not surprising, as they have been reported from multiple football academies using skeletal maturity [33, 34, 35]. It was beyond the scope of our study to examine a potential maturity selection bias; however, this possibility should be taken into consideration when interpreting our findings. Regardless, these observations are important to keep in mind as a coach or clinician working in age groups where players are at different stages of maturation and likely require a more individualised approach to performance development and injury risk management.

### Injury patterns differed depending on sexual maturity status

The overall injury incidence in this sample was relatively low (2.2 injuries per 1000 hours) compared to other studies of high-level youth football, where estimates of 5.8 per 1000 hours [36] and 5.7 per 1000 hours [37] have been reported in recent systematic reviews. We did, however, observe an increase in injury incidence in the older age groups. This trend is commonly observed in studies conducted at football academies [36, 37] and could potentially be attributed to players being bigger and more powerful, or to an increased training/ match load [38]. As in our study, some authors have found the highest incidence in U15-16 age groups [39, 40] linking this to the adolescent growth spurt and the phase during or after the age of peak height velocity (PHV). This would further coincide with most of the players in the U16 age group being in G4-5, which would indicate that they were either experiencing or had passed PHV [32, 41].

When analysing players by sexual maturity status, an increased injury incidence and burden were observed for players with more advanced maturity. This was consistent for all classification methods, where players in G4-5, PH5-6 and TV4 sustained a significantly higher number of injuries and lost more days, relative to playing time, compared to their less mature counterparts. As a larger proportion of players with advanced maturity status would be represented in the older age groups, it is not clear to what extent biological maturity *per se* influences injury risk, but it is an indication that it could be one factor that is relevant to consider when designing training or injury risk-mitigation programmes for adolescent players. Indeed, studies elsewhere have indicated that biological maturity status may affect the overall injury risk [42, 43], and others find no difference in overall incidence [35, 44]. When adjusted for age, point estimates in our study were still higher for more advanced pubertal stages; however, these differences were no longer significant.

Location- and type-specific injury incidence differed between age groups and maturity stages. Specifically, injuries to the ankle, knee, thigh and pelvis/groin were more common at the higher oldest team and in players more advanced in maturity. These players were also more likely to sustain muscle injuries, joint sprains, tendon injuries and haematomas/bruising. Conversely, “growth-related injuries” were the most common in the age groups U14 and U16, and among players at G3 and PH3 (i.e., mid-puberty). This supports the idea that different tissues may be more susceptible to injury depending on an athlete’s maturity status, with the attachment sites to the bone (i.e., apophyses) being relatively weak prior to skeletal maturity when muscles and tendons are more likely to sustain injuries due to acute incidents or overuse [45]. This changing pattern in location and type has not previously been reported for football players using indicators of sexual maturity, but is in concordance with studies applying other maturity indicators or examining differences between age groups [4]. For example, Monasterio et al. [46] noted that growth-related injuries, on average, were more likely to occur during PHV, whilst muscle, knee joint and ankle joint injuries were more frequent later in the maturation process. Their findings of a distal-to-proximal injury pattern for growth-related injuries [46] linked to the distinct sequence of maturation and growth spurts of different bones seen in non-athletic populations [1, 47] may also explain the differences in injury location observed in our sample.

When adjusting comparisons between maturity stages for age, only one significant difference was observed, with joint sprains being more common in players classified as PH6 compared to PH2. This might be expected, as maturity and age are related factors. Some authors hypothesise that combining data relating to training/match intensity and maturity may better explain the occurrence of injuries [48]. Indeed, both intensity and injury incidence typically increase as players get older, and although our analyses aimed to account for this by adjusting for age, more detailed investigations with larger samples (where robust comparisons of groups can be made within age groups) are required to understand which specific maturity-related factors (e.g., maturation stage of specific structures) contribute to the changing injury patterns and which are related to the increasing training and match demands in older age groups.

### Methodological considerations

Our study is novel in the sense that sexual maturity assessments from routine medical screenings were conducted during three seasons where a system was in place for club physicians to capture and classify injuries using a consistent and consensus-recommended injury surveillance system, including individual player exposure. Still, it is important to recognise some limitations, such as the invasive nature of sexual maturity staging (making it mainly applicable to clinical settings), inability to accurately determine the entry to a stage, limited use prior to and after adolescence, and potential variation in the precise distinction of specific stages [49]. There are, however, benefits of using this approach, including the close link to hormonal levels indicating the onset and end of puberty [5] and their clinical role in ruling out medical conditions (e.g., cryptorchidism, testicular agenesis, varicocele, hydrocele) during annual paediatric screenings [11–50]. It is also a cheap, fast and reproducible method for assessing maturity status. Still, it is important to highlight that variability can arise depending on the expertise of the clinician, particularly when comparing evaluations of experienced pediatricians or endocrinologists with those of other healthcare professionals. Although we unfortunately did not have specific intra-rater reliability data available for our study, assessments were conducted by a pediatrician with over ten years of experience in pediatric sports medicine and maturity assessments.

We were limited to one assessment per year, meaning that the maturity status at the start of the season may not perfectly align with injuries occurring later in the season. We also recognise that a timeloss injury definition is not sensitive to capture injuries that do not result in time away from training but may still cause pain or decrease performance. In future studies, it would also be worthwhile exploring other dimensions of training load (e.g., intensity) in addition to training duration. These factors could potentially have affected our observed injury incidence, which was relatively low compared to other studies. It is important to keep in mind that our sample was homogeneous, with the same developmental environment within a specific geographical location, including similar climate, training methodology and nutritional follow-up, as dictated by club policies. Finally, we acknowledge that injuries occur as a result of multiple interacting factors and encourage readers to not interpret our findings considering our observational study design, which is primarily descriptive and cannot establish any cause-and-effect relationships.

### Practical implications

It is important to highlight that the data on sexual maturity status in our study were not collected specifically to determine the injury risk of players, and we do not advocate that this should be implemented broadly on the basis of our findings. We do, however, believe that our study has used a more valid indicator of maturity status than has been the case in many previous studies, which have examined associations between maturity status and injury, and our findings therefore contribute to an improved understanding of this relationship. Where such assessments are already in place as part of routine medical screenings, our observations suggest that this information may provide contextual information which can be used to determine which injuries players are most likely to sustain (i.e., body part, type). This can be particularly useful in age groups where there is a wide variety in individuals’ maturity status. Assessments of maturity status are indeed common in professional academies, and there are indications that injury rates may be lowered by altering training during specific phases of the growth and maturity process [51]. An indication of sexual maturity may serve as an early indication that a player is entering the adolescent growth spurt [32], and preventative measures can be introduced accordingly. Similarly, knowledge about an athlete’s pubertal status may be used to guide training programmes aimed at athletic development [23]. Whilst regular monitoring of growth and maturity can be considered, to optimise players’ health and performance, this should be complemented with educational programmes for coaches, parents, athletes, practitioners and clinicians who are working with youth athletes [52].

## CONCLUSIONS

The novelty of this research lies in the use of sexual maturity indicators to assess maturation within a sports context, offering valuable insights for tailoring athletic development and injury management strategies. In a sample of Spanish male academy football players, we observed that injuries were more common and burdensome for more mature players. Players of advanced maturity also sustained more thigh, knee, ankle and pelvis/groin injuries, and more muscle injuries, joint sprains, tendon injuries and haematomas/bruising compared to less mature players. Conversely, growth-related injuries were more common during mid-puberty. Our observations are important for coaches and clinicians to consider when designing training and injury prevention programmes, as they suggest that different players within groups based primarily on chronological age may be susceptible to different injuries depending on their maturity status.

## Supplementary Material

Injuries according to sexual maturity status: a three-season observational study with male academy players of a professional Spanish football club

## References

[1] Malina RM, Bouchard C, Bar-Or O. Growth, maturation, and physical activity. 2nd ed. Champagne, IL: Human Kinetics; 2004.

[2] Swain M, Kamper SJ, Maher CG, Broderick C, McKay D, Henschke N. Relationship between growth, maturation and musculoskeletal conditions in adolescents: a systematic review. Br J Sports Med. 2018; 52(19):1246–1252. doi: 10.1136/bjsports-2017-098418. 29559438

[3] Wik EH. Growth, maturation and injuries in high-level youth football (soccer): A mini review. Front Sports Act Living. 2022; 4:975900. Published 2022 Nov 1. doi: 10.3389/fspor.2022.975900. 36385783 PMC9663653

[4] Parry GN, Williams S, McKay CD, Johnson DJ, Bergeron MF, Cumming SP. Associations between growth, maturation and injury in youth athletes engaged in elite pathways: a scoping review. Br J Sports Med. 2024; 58(17):1001–1010. Published 2024 Sep 4. doi: 10.1136/bjsports-2024-108233. 39209526 PMC11420720

[5] Wolf RM, Long D. Pubertal Development. Pediatr Rev. 2016; 37(7):292–300. doi: 10.1542/pir.2015-0065. 27368360

[6] Drobnic F, Albanell M, Pi R, Til L, Hernández G, Mónaco M. Guía de Revisión Médica del Futbol Club Barcelona. Certificado de Aptitud Deportiva. Barcelona: FC Barcelona; 2011.

[7] Tanner JM. Growth at adolescence. 2nd ed. Oxford, UK: Blackwell; 1962.

[8] Malina RM, Beunen G, Wellens R, Claessens A. Skeletal maturity and body size of teenage Belgian track and field athletes. Ann Hum Biol. 1986; 13(4):331–339. doi: 10.1080/03014468600008511. 3767306

[9] Matsudo SMM, Matsudo VKR. Self-assessment and physician assessment of sexual maturation in Brazilian boys and girls: Concordance and reproducibility. Am J Hum Biol. 1994; 6(4):451–455. doi: 10.1002/ajhb.1310060406. 28548259

[10] Huang B, Hillman J, Biro FM, Ding L, Dorn LD, Susman EJ. Correspondence Between Gonadal Steroid Hormone Concentrations and Secondary Sexual Characteristics Assessed by Clinicians, Adolescents, and Parents. J Res Adolesc. 2012; 22(2):381–391. doi: 10.1111/j.1532-7795.2011.00773.x. 23204809 PMC3507459

[11] Mónaco M, Verdugo F, Bodell M, Avendaño E, Til L, Drobnic F. Prevalencia de anomalías genitales en futbolistas jóvenes [Prevalence of genital anomalies in young football players]. An Pediatr (Barc). 2015; 82(1):e181–e183. doi: 10.1016/j.anpedi.2014.07.007. 25434530

[12] Mónaco M, Sanz Lopez F, Gutiérrez Rincón JA, Montoro Ronsano JB, Ibañez Toda L, Rodas G. Prospective study of maturation and injury in elite handball academy. Could ‘maturational status’ be a risk factor for injury incidence in different handball team categories? Apunts Sports Med. 2024; 59(221):100433. doi: 10.1016/j.apunsm.2023.100433.

[13] Etzel CM, Meghani O, Owens BD, Kocher MS, Field AE. Predictors of Anterior Cruciate Ligament Tears in Adolescents and Young Adults. Orthop J Sports Med. 2024; 12(9):23259671241272699. Published 2024 Sep 19. doi: 10.1177/23259671241272699. 39345931 PMC11439179

[14] Janssens KA, Rosmalen JG, Ormel J, et al. Pubertal status predicts back pain, overtiredness, and dizziness in American and Dutch adolescents. Pediatrics. 2011; 128(3):553–559. doi: 10.1542/peds.2010-2364. 21807699 PMC3164091

[15] Chevalley T, Bonjour JP, van Rietbergen B, Ferrari S, Rizzoli R. Fractures during childhood and adolescence in healthy boys: relation with bone mass, microstructure, and strength. J Clin Endocrinol Metab. 2011; 96(10):3134–3142. doi: 10.1210/jc.2011-1445. 21795454

[16] Mónaco M, Rincón JAG, Ronsano BJM, Whiteley R, Sanz-Lopez F, Rodas G. Injury incidence and injury patterns by category, player position, and maturation in elite male handball elite players. Biol Sport. 2019; 36(1):67–74. doi: 10.5114/biolsport.2018.78908. 30899141 PMC6413568

[17] Linder MM, Townsend DJ, Jones JC, Balkcom IL, Anthony CR. Incidence of adolescent injuries in junior high school football and its relationship to sexual maturity. Clin J Sport Med. 1995; 5(3):167–170. doi: 10.1097/00042752-199507000-00006. 7670972

[18] Caine D, Cochrane B, Caine C, Zemper E. An epidemiologic investigation of injuries affecting young competitive female gymnasts. Am J Sports Med. 1989; 17(6):811–820. doi: 10.1177/036354658901700616. 2696378

[19] Tenforde AS, Sayres LC, McCurdy ML, Sainani KL, Fredericson M. Identifying sex-specific risk factors for stress fractures in adolescent runners. Med Sci Sports Exerc. 2013; 45(10):1843–1851. doi: 10.1249/MSS.0b013e3182963d75. 23584402

[20] Decloe MD, Meeuwisse WH, Hagel BE, Emery CA. Injury rates, types, mechanisms and risk factors in female youth ice hockey. Br J Sports Med. 2014; 48(1):51–56. doi: 10.1136/bjsports-2012-091653. 23446642

[21] Rauh MJ, Nichols JF, Barrack MT. Relationships among injury and disordered eating, menstrual dysfunction, and low bone mineral density in high school athletes: a prospective study. J Athl Train. 2010; 45(3):243–252. doi: 10.4085/1062-6050-45.3.243. 20446837 PMC2865962

[22] Dut R, Akgül S, Dönmez G, Ulkar B, Kanbur N, Derman O. Adolescent male soccer players have higher growth rates and risk of injury is associated with biological maturity. Turk J Pediatr. 2023; 65(6):990–1001. doi: 10.24953/turkjped.2023.140. 38204314

[23] Towlson C, Salter J, Ade JD, et al. Maturity-associated considerations for training load, injury risk, and physical performance in youth soccer: One size does not fit all. J Sport Health Sci. 2021; 10(4):403–412. doi: 10.1016/j.jshs.2020.09.003. 32961300 PMC8343060

[24] Catalunya Gd. Protocol d’activitats preventives i de promoció de la salut a l’edat pediàtrica. Barcelona In: Salut Dd, ed: Generalitat de Catalunya; 2008.

[25] Physicians AAoF, Pediatrics AAo, Medicine ACoS, Medicine AMSfS, Medicine AOSfS, Medicine AOAoS. Preparticipation Physical Evaluation. American Academy of Pediatrics; 2019.

[26] Rosen DS. Physiologic growth and development during adolescence. Pediatr Rev. 2004; 25(6):194–200. doi: 10.1542/pir.25-6-194. 15173452

[27] Prader A. Testicular size: assessment and clinical importance. Triangle. 1966; 7(6):240–243. 5920758

[28] Fuller CW, Ekstrand J, Junge A, et al. Consensus statement on injury definitions and data collection procedures in studies of football (soccer) injuries. Br J Sports Med. 2006; 40(3):193–201. doi: 10.1136/bjsm.2005.025270. 16505073 PMC2491990

[29] Rae K, Orchard J. The Orchard Sports Injury Classification System (OSICS) version 10. Clin J Sport Med. 2007; 17(3):201–204. doi: 10.1097/JSM.0b013e318059b536. 17513912

[30] Gail MH, Benichou J. Encyclopedia of epidemiologic methods. John Wiley & Sons; 2000.

[31] Frome EL, Checkoway H. Epidemiologic programs for computers and calculators. Use of Poisson regression models in estimating incidence rates and ratios. Am J Epidemiol. 1985; 121(2):309–323. doi: 10.1093/oxfordjournals.aje.a114001.32. 3839345

[32] Marshall WA, Tanner JM. Variations in the pattern of pubertal changes in boys. Arch Dis Child. 1970; 45(239):13–23. doi: 10.1136/adc.45.239.13. 5440182 PMC2020414

[33] Wik EH, Chamari K, Tabben M, Di Salvo V, Gregson W, Bahr R. Exploring Growth, Maturity, and Age as Injury Risk Factors in High-Level Youth Football. Sports Med Int Open. 2024; 8:a21804594. Published 2024 Jan 8. doi: 10.1055/a-2180-4594. 38312925 PMC10832576

[34] Monasterio X, Gil SM, Bidaurrazaga-Letona I, et al. The burden of injuries according to maturity status and timing: A two-decade study with 110 growth curves in an elite football academy. Eur J Sport Sci. 2023; 23(2):267–277. doi: 10.1080/17461391.2021.2006316. 34767492

[35] Le Gall F, Carling C, Reilly T. Biological maturity and injury in elite youth football. Scand J Med Sci Sports. 2007; 17(5):564–572. doi: 10.1111/j.1600-0838.2006.00594.x. 17076832

[36] Jones S, Almousa S, Gibb A, et al. Injury Incidence, Prevalence and Severity in High-Level Male Youth Football: A Systematic Review. Sports Med. 2019; 49(12):1879–1899. doi: 10.1007/s40279-019-01169-8. 31452129

[37] Robles-Palazón FJ, López-Valenciano A, De Ste Croix M, et al. Epidemiology of injuries in male and female youth football players: A systematic review and meta-analysis. J Sport Health Sci. 2022; 11(6):681–695. doi: 10.1016/j.jshs.2021.10.002. 34700052 PMC9729930

[38] Buchheit M, Mendez-Villanueva A, Simpson BM, Bourdon PC. Match running performance and fitness in youth soccer. Int J Sports Med. 2010; 31(11):818–825. doi: 10.1055/s-0030-1262838. 20703978

[39] Bult HJ, Barendrecht M, Tak IJR. Injury Risk and Injury Burden Are Related to Age Group and Peak Height Velocity Among Talented Male Youth Soccer Players. Orthop J Sports Med. 2018; 6(12):2325967118811042. Published 2018 Dec 11. doi: 10.1177/2325967118811042. 30560140 PMC6293374

[40] Materne O, Chamari K, Farooq A, et al. Injury incidence and burden in a youth elite football academy: a four-season prospective study of 551 players aged from under 9 to under 19 years. Br J Sports Med. 2021; 55(9):493–500. doi: 10.1136/bjsports-2020-102859. 33199359

[41] Granados A, Gebremariam A, Lee JM. Relationship Between Timing of Peak Height Velocity and Pubertal Staging in Boys and Girls. J Clin Res Pediatr Endocrinol. 2015; 7(3):235–237. doi: 10.4274/jcrpe.2007. 26831559 PMC4677560

[42] Materne O, Chamari K, Farooq A, et al. Association of Skeletal Maturity and Injury Risk in Elite Youth Soccer Players: A 4-Season Prospective Study With Survival Analysis. Orthop J Sports Med. 2021; 9(3):2325967121999113. Published 2021 Mar 31. doi: 10.1177/2325967121999113. 33869641 PMC8020116

[43] Monasterio X, Cumming SP, Larruskain J, et al. The combined effects of growth and maturity status on injury risk in an elite football academy. Biol Sport. 2024; 41(1):235–244. doi: 10.5114/biolsport.2024.129472. 38188110 PMC10765440

[44] Johnson A, Doherty PJ, Freemont A. Investigation of growth, development, and factors associated with injury in elite schoolboy footballers: prospective study. BMJ. 2009; 338:b490. Published 2009 Feb 26. doi: 10.1136/bmj.b490. 19246550 PMC2651105

[45] Brukner P, Khan KM. Brukner & Khan’s Clinical Sports Medicine. 4th ed. New York, NY: McGraw-Hill Medical; 2012.

[46] Monasterio X, Gil SM, Bidaurrazaga-Letona I, et al. Injuries according to the percentage of adult height in an elite soccer academy. J Sci Med Sport. 2021; 24(3):218–223. doi: 10.1016/j.jsams.2020.08.004. 32839106

[47] Kvist O, Luiza Dallora A, Nilsson O, et al. A cross-sectional magnetic resonance imaging study of factors influencing growth plate closure in adolescents and young adults. Acta Paediatr. 2021; 110(4):1249–1256. doi: 10.1111/apa.15617. 33047349 PMC7983983

[48] Costa e Silva L, Silva AL, Teles J, Fragoso I. Study on the Influence of Physical Activity Intensity and Maturation on Sports Injuries in Children and Adolescents. Applied Sciences. 2024; 14(22):10632. 10.3390/app142210632.

[49] Beunen GP, Rogol AD, Malina RM. Indicators of biological maturation and secular changes in biological maturation. Food Nutr Bull. 2006; 27(4 Suppl Growth Standard):S244–S256. doi: 10.1177/15648265060274S508. 17361661

[50] Dias Filho AC, Cruz PRCD, Ribeiro PRF, Riccetto CLZ. Testicular volume and Tanner stage: determinant factors for testicular torsion. Einstein (Sao Paulo). 2022; 20:eAO6605. Published 2022 Apr 20. doi: 10.31744/einstein_journal/2022AO6605. 35476083 PMC9000982

[51] Johnson D, Williams S, Bradley B, Cumming SP. Can we reduce injury risk during the adolescent growth spurt? An iterative sequence of prevention in male academy footballers. Ann Hum Biol. 2023; 50(1):452–460. doi: 10.1080/03014460.2023.2261854. 37823577

[52] Bergeron MF, Côté J, Cumming SP, et al. IOC consensus statement on elite youth athletes competing at the Olympic Games: essentials to a healthy, safe and sustainable paradigm. Br J Sports Med. 2024; 58(17):946–965. Published 2024 Sep 4. doi: 10.1136/bjsports-2024-108186. 39197945

