# Supplementary material for: Injuries according to sexual maturity status: a three-season observational study with male academy players of a professional Spanish football club
Source: Biol Sport. 2025 Sep 29;43:393–403. doi: 10.5114/biolsport.2026.154143 (PMC12884892; doi:10.5114/biolsport.2026.154143)
Supplement: Injuries according to sexual maturity status: a three-season observational study with male academy players of a professional Spanish football club [file JBS-43-56662-s1.pdf]

SUPPLEMENTARY MATERIAL

SUPPLEMENTARY TABLE 1. Age-adjusted injury incidence (injuries per 1000 h, 95% CI) for the most relevant injury locations in a Spanish male football academy, grouped by sexual maturity status.

	Foot	Ankle	Lower leg	Knee	Thigh	Pelvis & Groin	Wrist & Hand	Head
G1	0.2 (0.1–0.5)	0.2 (0.1–0.5)	0.2 (0.1–0.5)	0.3 (0.2–0.6)	0.2 (0.1–0.5)	0.4 (0.2–0.8)	0.1 (0.0–0.3)	0.1 (0.0–0.3)
G2	0.0 (0.0–0.0)	0.1 (0.0–0.5)	0.0 (0.0–0.0)	0.3 (0.1–0.8)	0.3 (0.1–0.7)	0.2 (0.1–0.7)	0.3 (0.1–0.7)	0.1 (0.0–0.5)
G3	0.1 (0.0–0.4)	0.1 (0.0–0.4)	0.1 (0.0–0.4)	0.2 (0.1–0.5)	0.2 (0.1–0.7)	0.4 (0.2–0.9)	0.3 (0.1–0.7)	0.1 (0.0–0.4)
G4	0.2 (0.1–0.6)	0.5 (0.2–1.0)	0.0 (0.0–0.0)	0.2 (0.1–0.6)	0.4 (0.1–0.8)	0.4 (0.2–0.9)	0.1 (0.0–0.6)	0.0 (0.0–0.0)
G5	0.2 (0.0–0.6)	0.5 (0.2–1.1)	0.1 (0.0–0.3)	0.2 (0.1–0.6)	0.7 (0.3–1.5)	0.1 (0.0–0.3)	0.2 (0.1–0.8)	0.1 (0.0–0.4)
PH1	0.2 (0.1–0.4)	0.2 (0.1–0.4)	0.1 (0.1–0.4)	0.3 (0.1–0.6)	0.2 (0.1–0.5)	0.3 (0.2–0.6)	0.1 (0.0–0.3)	0.1 (0.0–0.3)
PH2	0.0 (0.0–0.0)	0.1 (0.0–0.8)	0.0 (0.0–0.0)	0.2 (0.1–0.9)	0.2 (0.1–0.8)	0.3 (0.1–1.0)	0.2 (0.1–0.8)	0.0 (0.0–0.0)
PH3	0.1 (0.0–0.5)	0.1 (0.0–0.5)	0.1 (0.0–0.4)	0.2 (0.1–0.5)	0.2 (0.1–0.6)	0.5 (0.2–0.9)	0.3 (0.1–0.8)	0.1 (0.0–0.4)
PH4	0.2 (0.1–0.6)	0.5 (0.3–1.0)	0.0 (0.0–0.0)	0.2 (0.1–0.5)	0.3 (0.1–0.8)	0.5 (0.2–0.9)	0.1 (0.0–0.5)	0.0 (0.0–0.3)
PH5	0.2 (0.0–0.6)	0.4 (0.2–0.9)	0.1 (0.0–0.4)	0.2 (0.1–0.6)	0.7 (0.3–1.6)	0.1 (0.1–0.4)	0.2 (0.0–0.7)	0.1 (0.0–04)
PH6	0.2 (0.0–1.4)	0.6 (0.2–1.6)	0.1 (0.0–0.6)	0.1 (0.0–0.6)	0.6 (0.2–1.7)	0.1 (0.0–0.5)	0.1 (0.0–1.2)	0.1 (0.0–0.7)
TV1	0.2 (0.1–0.4)	0.2 (0.1–0.5)	0.1 (0.0–0.4)	0.4 (0.2–0.7)	0.2 (0.1–0.5)	0.4 (0.2–0.7)	0.1 (0.0–0.2)	0.1 (0.0–0.3)
TV2	0.0 (0.0–0.0)	0.1 (0.0–0.5)	0.1 (0.0–0.5)	0.3 (0.1–0.8)	0.3 (0.1–0.7)	0.3 (0.1–0.7)	0.2 (0.1–0.5)	0.1 (0.0–0.5)
TV3	0.1 (0.0–0.3)	0.1 (0.0–0.4)	0.0 (0.0–0.3)	0.1 (0.1–0.4)	0.1 (0.0–0.4)	0.4 (0.2–0.8)	0.2 (0.1–0.6)	0.0 (0.0–0.0)
TV4	0.2 (0.1–0.6)	0.5 (0.2–1.0)	0.1 (0.0–0.3)	0.2 (0.1–0.5)	0.6 (0.3–1.2)	0.2 (0.1–0.5)	0.3 (0.1–0.9)	0.1 (0.0–0.5)

G1-5: Genital stage 1–5; PH1-6: Pubic hair stage 1–6; TV1-4: Testicular volume stage 1–4

SUPPLEMENTARY TABLE 2. Age-adjusted injury incidence (injuries per 1000 h, 95% CI) for the most relevant injury types in a Spanish male football academy, grouped by sexual maturity status.

	Muscle injury	Joint sprain	Growth-related	Fracture	Hematoma/Bruising	Tendon injury
G1	0.2 (0.1–0.4)	0.3 (0.2–0.6)	0.6 (0.3–1.0)	0.1(0.0–0.3)	0.2 (0.1–0.5)	0.0 (0.0–0.2)
G2	0.3 (0.1–0.8)	0.1 (0.0–0.6)	0.3 (0.1–0.8)	0.2 (0.1–0.6)	0.2 (0.1–0.7)	0.1 (0.0–0.5)
G3	0.3 (0.1–0.8)	0.1 (0.0–0.3)	0.7 (0.4–1.2)	0.4 (0.2–0.9)	0.1 (0.0–0.4)	0.1 (0.0–0.5)
G4	0.4 (0.2–1.0)	0.3 (0.2–0.7)	0.5 (0.2–0.9)	0.3 (0.1–0.8)	0.1 (0.1–0.4)	0.1 (0.0–0.6)
G5	0.9 (0.4–1.9)	0.3 (0.2–0.8)	0.1 (0.1–0.4)	0.3 (0.1–0.9)	0.0 (0.0–0.2)	0.4 (0.1–1.5)
PH1	0.2 (0.1–0.4)	0.3 (0.2–0.6)	0.4 (0.3–0.8)	0.2 (0.1–0.4)	0.2 (0.1–0.5)	0.0 (0.0–0.2)
PH2	0.2 (0.1–0.8)	0.0 (0.0–0.0)	0.4 (0.2–1.2)	0.2 (0.1–0.8)	0.2 (0.1–0.9)	0.1 (0.0–0.7)
PH3	0.3 (0.1–0.8)	0.1 (0.0–0.4)	0.8 (0.4–1.4)	0.3 (0.1–0.9)	0.1 (0.0–0.4)	0.1 (0.0–0.5)
PH4	0.3 (0.1–0.8)	0.3 (0.1–0.6)	0.5 (0.3–1.0)	0.3 (0.1–0.7)	0.2 (0.1–0.5)	0.1 (0.0–0.6)
PH5	1.1 (0.5–2.2)	0.3 (0.1–0.7)	0.2 (0.1–0.5)	0.2 (0.1–0.7)	0.0 (0.0–0.1)	0.4 (0.1–1.5)
PH6	0.2 (0.1–0.4)	0.3 (0.2–0.6) ^{2}	0.4 (0.3–0.8)	0.2 (0.1–0.4)	0.0 (0.0–0.2)	0.3 (0.1–1.9)
TV1	0.2 (0.1–0.4)	0.4 (0.2–0.7)	0.5 (0.3–0.9)	0.1 (0.0–0.3)	0.1 (0.0–0.3)	0.1 (0.0–0.3)
TV2	0.3 (0.1–0.7)	0.2 (0.1–0.6)	0.4 (0.2–0.9)	0.1 (0.0–0.5)	0.3 (0.1–0.7)	0.1 (0.0–0.5)
TV3	0.2 (0.1–0.5)	0.1 (0.0–0.3)	0.6 (0.3–1.0)	0.5 (0.2–1.0)	0.0 (0.0–0.3)	0.1 (0.0–0.4)
TV4	0.8 (0.5–1.5)	0.3 (0.2–0.6)	0.2 (0.1–0.5)	0.3 (0.1–0.7)	0.2 (0.1–0.5)	0.2 (0.1–0.5)

G1-5: Genital stage 1–5; PH1-6: Pubic hair stage 1–6; TV1-4: Testicular volume stage 1–4.
Characters in superscript indicate significant differences ($p < 0.05$) compared to other stages within the same group (^{1}Stage 1, ^{2}Stage 2, ^{3}Stage 3, ^{4}Stage 4, ^{5}Stage 5).

Articles published in the Biology of Sport are licensed under an open access Creative Commons CC BY 4.0 license.
